# Supplementary material for: Mechanisms governing the pioneering and redistribution capabilities of the non-classical pioneer PU.1
Source: Nat Commun. 2020 Jan 21;11:402. doi: 10.1038/s41467-019-13960-2 (PMC6972792; doi:10.1038/s41467-019-13960-2)
Supplement: Supplementary file 7 — Source data [file 41467_2019_13960_MOESM7_ESM.zip › Source_Data/Figure6/Figure6B_MotifScanOutput/ETS1_mutPU.1/homerResults/motif24.similar.html]

motif24

## Information for motif24

C
T
A
G
T
C
A
G
A
G
T
C
G
A
T
C
G
T
A
C
A
G
T
C
C
A
T
G
A
G
T
C
A
G
T
C
A
G
T
C
  
Reverse Opposite:  

C
T
A
G
A
C
T
G
A
C
T
G
G
T
A
C
A
C
T
G
C
A
T
G
C
T
A
G
T
C
A
G
A
G
T
C
G
A
T
C
  

|  |  |
| --- | --- |
| p-value: | 1e-95 |
| log p-value: | -2.193e+02 |
| Information Content per bp: | 1.658 |
| Number of Target Sequences with motif | 2557.0 |
| Percentage of Target Sequences with motif | 18.96% |
| Number of Background Sequences with motif | 4051.3 |
| Percentage of Background Sequences with motif | 11.55% |
| Average Position of motif in Targets | 100.9 +/- 56.5bp |
| Average Position of motif in Background | 97.6 +/- 62.9bp |
| Strand Bias (log2 ratio + to - strand density) | -0.0 |
| Multiplicity (# of sites on avg that occur together) | 1.12 |
| Motif File: | file (matrix) reverse opposite |

### Similar de novo motifs found

|  |  |  |  |  |  |  |  |
| --- | --- | --- | --- | --- | --- | --- | --- |
| Rank | Match Score | Redundant Motif | P-value | log P-value | % of Targets | % of Background | Motif file |
| 1 | 0.889 | T C A G C T A G C T A G A C T G A C T G G A T C C T A G C A T G C T A G T A C G T G A C A G T C C A G T | 1e-70 | -163.398359 | 11.61% | 6.54% | motif file (matrix) |
| 2 | 0.924 | C T A G T C A G A C T G G A T C A C T G A C T G C T A G T A C G G T A C G A T C C T G A T C G A | 1e-67 | -154.473958 | 9.74% | 5.24% | motif file (matrix) |
| 3 | 0.772 | A G T C A G T C A C T G A G T C A G T C G T A C A C G T | 1e-30 | -69.241156 | 8.36% | 5.46% | motif file (matrix) |
| 4 | 0.789 | T G C A T C G A T C A G A G T C G A T C A G T C G T A C C T A G A T G C A G T C A G T C G A T C G C A T A T G C | 1e-22 | -51.166433 | 1.55% | 0.58% | motif file (matrix) |
| 5 | 0.755 | A C T G A C T G A C T G C G T A A C T G A C G T G T C A A C T G | 1e-13 | -29.974981 | 2.16% | 1.22% | motif file (matrix) |
